# The use of TNF-α blockers in psoriatic arthritis patients with latent tuberculosis infection

**DOI:** 10.1007/s10067-014-2536-z

**Published:** 2014-02-21

**Authors:** Mariangela Atteno, Luisa Costa, Alessandro Matarese, Francesco Caso, Antonio Del Puente, Luca Cantarini, Maria Luisa Bocchino, Alessandro Sanduzzi, Raffaele Scarpa

**Affiliations:** 1 Department of Clinical and Experimental Medicine, Rheumatology Unit, University Federico II, Via S. Pansini 5, 80131 Naples, Italy; 2 Department of Clinical and Experimental Medicine, Respiratory Research Units, University Federico II, Naples, Italy; 3 Department of Medicine DIMED, Rheumatology Unit, University of Padova, Padova, Italy; 4 Research Center of Systemic Autoimmune and Autoinflammatory Diseases, Rheumatology Unit, Policlinico Le Scotte, University of Siena, Siena, Italy

**Keywords:** Anti-TNF-α, IGRA, LTBI, PsA, TST, Tuberculosis

## Abstract

Psoriatic arthritis (PsA) is an inflammatory arthropathy associated with skin and/or nail psoriasis. TNF-α is an essential cytokine for the host defense, and its depletion by treatment may facilitate the risk of infections or their reactivation. The aim of this study was to evaluate the efficacy and safety of TNF-α blockers in patients with PsA and concomitant latent tuberculosis infection (LTBI) comparing their outcome with non-infected PsA patients. This is a retrospective study in 321 patients with PsA, attending the Psoriatic Arthritis Clinic at the University Federico II of Naples, who had an inadequate response to DMARDs and started therapy with TNF-α blockers. We identified 40 patients with LTBI, who were included in this study along with 40 not infected PsA patients as control group. At baseline (T0) and every 3 months for 2 years (T2), data concerning PsA activity were registered. All patients underwent chest X-ray every 6 months (or 12 if appropriate). In each group, 22 patients were on etanercept therapy, 14 on adalimumab, and 4 on infliximab. Anti-TNF-α therapy was effective in both group of patients, and no statistically significant differences were found in the analysis of the study variables between the two groups from T0 to T2. No serious adverse events occurred in both groups, and no patient was withdrawn from therapy. Our experience suggests that anti-TNF-α treatment is effective and safe in PsA patients with concomitant LTBI. Therefore, neither LTBI nor chemoprophylaxis seems to influence the course of anti-TNF-α therapy.

## Introduction

Psoriatic arthritis (PsA) is an inflammatory arthropathy, associated with skin and/or nail psoriasis [1–3] and other possible systemic features [4–6].

In the last decade, the introduction of biologic drugs, in particular tumor necrosis factors (TNF)-α blockers, has opened new horizons for patients and rheumatologists in the treatment of PsA and other rheumatic diseases [7–10].

The use of these agents has outlined the importance of evaluating their safety, as it may expose patients to an increased risk of developing infections, in particular tuberculosis (TB) [11].

The global prevalence of TB is still close to 9 million, mainly in underdeveloped countries. This condition is believed to affect one third of the world’s population, with a natural history showing a progression to active disease in only 5 % of cases [12].

TNF-α is an essential pro-inflammatory cytokine also involved in the host defense against *Mycobacterium tuberculosis* (MTB) [13, 14]. Therefore, patients eligible for anti-TNF-α therapy require careful evaluation and need to be investigated about possible previous exposure to MTB, as its use may expose patients to an increased risk of developing active TB and reactivation of latent tuberculosis infection (LTBI) [15].

The aim of this study was to evaluate the efficacy and safety of TNF-α blockers in patients with PsA and concomitant LTBI comparing their outcome with non-infected PsA patients.

## Patients and methods

We performed a retrospective study, from January 2005 to December 2011, in 321 Caucasian patients with PsA, with no specific exposure risk to TB, attending the Psoriatic Arthritis Clinic at the University Federico II of Naples, who had an inadequate response to disease-modifying antirheumatic drugs (DMARDs) and started therapy with TNF-α blockers.

Before starting anti-TNF-α therapy, all patients, according to our screening protocol [15], undergo clinical medical history, physical examination, laboratory standard tests, chest X-ray, and tuberculin skin test (TST). Patients, not vaccinated, with a positive TST are considered affected by LTBI. Therefore, they are not immediately eligible for TNF-α blocker therapy and have to start treatment for LTBI before starting biologic therapy. Negative TST patients, if not treated with immunosuppressive drugs or steroids and if immunocompetent, started TNF-α blocker therapy at time of enrollment, performing an INF-γ release assay (IGRA) every 12 months.

In case of negative patients under immunosuppressive drugs or prolonged steroid therapy or positive patients previously vaccinated, we perform in addition an IGRA test.

Treatment for LTBI consists of a 9-month therapy with isoniazid monitoring adverse events, in particular liver function tests, monthly. Anti-TNF-α treatment was always started after the first 45 days of antitubercular therapy.

We identified 40 patients with LTBI, who were included in this study along with 40 not infected PsA patients as control group, matched for age, sex, and disease duration. For all patients included in the study, we collected the following data at starting anti-TNF-α therapy (T0) and every 3 months for the 2 years (T2) of follow up: physical examination, recording of vital signs, tender joint count (TJC; 68 tender joints), swollen joint count (SJC; 66 swollen joints), Health Assessment Questionnaire (HAQ), Psoriasis Area and Severity Index (PASI), visual analogic scale (VAS), erythrocyte sedimentation rate (ESR), and C-reactive protein (CRP). All patients underwent chest X-ray every 6 months (or 12 if appropriate).

### Tuberculin skin test

Two units (0.1 ml) of standard preparation of PPD RT-23 (Statens Serum Institut, Copenhagen, Denmark) were injected in the intradermal region of the forearm volar surface (Mantoux method). The reaction was read at 72 h with the transverse diameter in millimeters of induration.

The cutoff for a positive skin test was defined as an induration area greater than or equal to 5 mm in diameter.

### QuantiFERON-TB Gold In-Tube

QuantiFERON-TB Gold In-Tube (QFT-GIT) test used designed (1 mL) blood collection tubes that were coated with *M. tuberculosis*-specific antigens [ESAT-6, CFP-10, and TB 7.7(p4)], along with a negative and a positive control tube. An enzyme-linked immunosorbent assay (ELISA) was used to measure the amount of IFN-γ present in each of the three tubes (Nil control, TB-Antigen, Mitogen control).

### Statistical analysis

Statistical analysis was performed using SPSS software (SPSS Inc., Chicago, IL, USA). Descriptive statistics included mean values and standard deviation (SD) of the continuous variables and percentages and proportions of the categorical variables. For the variables of interest, a difference (delta) between T2 and T0 was computed, and Student’s test (for paired samples) was used for the analysis.

To compare continuous variables and dichotomous ones, Mann-Whitney and *χ* ^2^ tests were performed, respectively.

Correlations between study variables have been calculated using analysis of covariance (ANCOVA) and logistic regression. For logistic regression, different methods were used for the selection of variables (full model, backward, stepwise) to exclude the influence of the different methodologies. To check the effects of possible confounding variables, which can affect TST results, we computed the correlation coefficients, checking the specific effects of the different variables, as described successively.

In the ANCOVA, we compared values and changes (at T0 and T2) in CRP, ESR, VAS, TJC, SJC, HAQ, and PASI between the two groups, adjusting for the following variables: type and duration of therapy with anti-TNF; type, number, and duration of concomitant DMARD; use and duration of concomitant steroids; and use and duration of isoniazid.

## Results

On the basis of our screening, 40 patients (12.5 %) (group 1) were diagnosed to have LTBI (12 males and 28 females, mean age 51.6 ± 12 years). All patients had a negative history of contacts and chest X-ray negative for TB. Among 281 not infected patients, we selected 40 consecutive patients (group 2) (12 males and 28 females, mean age 51.4 ± 11.9 years), who used the TNF-α blockers for the entire 2-year period of observation, matched for gender, age, disease duration, and type of TNF-α blocker.

Demographic and clinical characteristics at baseline of both groups selected for this study were reported in Table 1.

**Table 1:** Demographic and clinical characteristic of two groups of patients at baseline

	Group 1	Group 2
Number	40	40
Female, *n*	28	28
Age (years)	51.6 ± 12	51.4 ± 11.9
Disease duration (months)	58.44 ± 35.28	59.4 ± 33.6
CRP (mg/dl)	0.9 ± 1.2	0.4 ± 0.2
ESR (mm/hr)	23.1 ± 20.3	15.7 ± 11.5
VAS	78.8 ± 11.6	76.2 ± 10.1
HAQ	1.5 ± 0.6	1.9 ± 0.5
TJC	12.9 ± 4.7	17.1 ± 7.6
SJC	2.7 ± 2	2.6 ± 3.1
PASI	0.77 ± 0.89	0.68 ± 0.76

Data expressed as mean ± standard deviation, unless otherwise indicated

*CRP* C-reactive protein, *ESR* erythrocyte sedimentation rate, *VAS* visual analogic scale, *HAQ* Health Assessment Questionnaire, *TJC* tender joint count, *SJC* swollen joint count, *PASI* Psoriasis Area and Severity Index

At recruitment, patients of group 1 had used steroids for a mean period of time greater than patients of group 2 (10.8 ± 6.4 vs 6.3 ± 4.8 months; *p* = 0.32). In detail, steroid therapy had been used in 26 patients of group 1 and 24 of group 2.

Moreover, the mean duration of DMARDs treatment was greater in group 2 vs group 1 (41.7 ± 40.8 vs 24.5 ± 38.6 months; *p* = 0.21). In group 1, a total of 11 (28 %) patients was taking immunosuppressive therapy (cyclosporine *n* = 1, methotrexate *n* = 7, leflunomide *n* = 1, salazopyrin *n* = 2). In group 2, a total of 15 (38 %) patients was taking immunosuppressive therapy (cyclosporine *n* = 1, methotrexate *n* = 11, leflunomide *n* = 1, salazopyrin *n* = 2).

No statistically significant differences were found in the comparison between the two groups.

There was no significant correlation between the TST result and previous therapies with steroids and DMARDs (odds ratio 1.73, IC 0.53–6.57).

### Effectiveness and safety of anti-TNF-α

Patients of group 1 started anti-TNF-α therapy after at least 45 days on prophylactic therapy with isoniazid. Patients of group 2, who started anti-TNF-α therapy, performed IGRA test every 12 months to detect a possible LTBI occurrence.

In each group, 22 patients were on etanercept therapy, 14 on adalimumab, and 4 on infliximab.

Anti-TNF-α therapy was effective in both groups of patients, and no statistically significant differences were found in the analysis of the study variables between the two groups from T0 to T2 (Table 2).

**Table 2:** Comparison of variation of clinical characteristic in both groups of patients at T0 and T2

	Group 1	Group 2	*P*
CRP (mg/dl)	0.306	0.081	0.6085
ESR (mm/hr)	0.362	0.034	0.9609
VAS	0.0001	0.0001	0.2509
HAQ	0.0001	0.0001	0.2593
TJC	0.0001	0.0001	0.1772
SJC	0.017	0.0001	0.8446
PASI	0.0001	0.0001	0.3531

*CRP* C-reactive protein, *ESR* erythrocyte sedimentation rate, *VAS* visual analogic scale, *HAQ* Health Assessment Questionnaire, *TJC* tender joint count, *SJC* swollen joint count, *PASI* Psoriasis Area and Severity Index

No serious adverse events occurred in both groups, and no patient was withdrawn from therapy. During the follow-up period, all patients on steroid reduced gradually and discontinued steroid within the first 3 months, while DMARDs therapy was continued for the entire 2-year period.

Among patients of group 1, no one developed active TB during follow-up, while two patients of group 2 showed positive IGRA results after 1 year of treatment. In those cases, biologic therapy was temporally stopped and isoniazid therapy was started. After 45 days, no signs of active MTB infection occurred and biologic therapy was restarted.

## Discussion

This paper reports our experience in PsA patients with concomitant LTBI under anti-TNF-α drugs. This topic is still substantially underestimate in the literature. However, given the epidemiological dynamics, it may represent an emerging question which we need to face.

The results of this study show that the presence of concomitant LTBI in PsA patients does not influence the effectiveness of anti-TNF-α therapy during the follow-up period. In fact, TNF-α blockers, as expected, improved signs of articular and cutaneous involvement and ameliorated function and quality of life in patients of both groups after 24 months of therapy.

Further important result concerns safety: there were no cases of active tuberculosis in both groups during the follow-up period.

Although anti-TNF-α agents are effective in the treatment of PsA, their use has been recognized as a risk factor for active tuberculosis development in patients with immune-mediated inflammatory diseases, including rheumatoid arthritis (RA), ankylosing spondylitis (AS), Crohn’s disease, PsA, and psoriasis [16, 17]. The mechanism by which TNF-α antagonists reactivate latent TB is not fully understood. In fact, TNF-α is an essential cytokine for the host’s defense against infective pathogens [14]. It has shown that during MTB infection, TNF-α is produced transiently and focally by granulomas in response to mycobacterial challenge [13]. It contributes to their containment and elimination, suggesting a pivotal role of TNF-α in defense mechanisms against microorganisms [13].

In animal models, TNF-α plays a central role in mediating mycobacterial infections, and soluble TNF (especially membrane-bound TNF) expressed by activated macrophages and T lymphocytes is essential in protecting against TB infection [18].

It has been observed that most cases of TB develop soon after treatment starting and correspond to reactivation of LTBI [19, 20]. Several recommendations for screening patients with LTBI infection and for the treatment of such patients before initiating anti-TNF-α have been proposed, even if at the present there are no international guidelines for monitoring of LTBI during biologic treatment and the data are poor [15].

According to these recommendations, several studies have shown that isoniazid therapy for 6 and 12 months in patients with LTBI is more effective than placebo in preventing reactivation, and the best benefit can be obtained with 9 months of therapy [21]. In our population, none of the patients treated with isoniazid developed serious toxicity in the liver, but it remains a threat that should be considered, especially in the event of concomitant use of hepatotoxic drugs other than isoniazid or methotrexate [22, 23]. Effectiveness of chemoprophylaxis is limited not only by the efficacy of the drug but also by patient adherence to therapy.

A recent open-label study tested the efficacy and the tolerability of a 3-month regimen with isoniazid plus rifampicin, showing that this therapy could be useful in patients with rheumatic conditions proposed for anti-TNF therapy [24].

The French Research Axed on Tolerance of Biotherapies (RATIO) registry records the annual incidence rate of TB in patients treated with anti-TNF-α blockers, adjusted for age and sex, with the French population used as reference [25]. Data from this registry report 69 cases of active TB in patients with anti-TNF-α for a period of 3 years for a total of 57,711 patient-years [25]. TB cases were related to infliximab and adalimumab therapy, and the authors underline that none of the patients had received correct LTBI chemoprophylaxis according to the French recommendations [25].

The British registry confirms these data, underlining a relative risk of TB three to four times higher for the monoclonal antibodies than for etanercept. In addition, the data show a greater increased risk of extrapulmonary disease with the monoclonal antibodies [26].

In our study we did not observe any case of reactivation of TB in patients with LTBI treated with TNF-α blockers. This result may be affected by the relative small sample size. On the other hand, it should be considered screening, chemoprophylaxis, and monitoring of patients [15], which may explain this optimal result. In fact, the question of compliance to therapy seems to be critical. Current evidence indicates that prophylaxis may be ineffective in up to 30 % of cases, when patient adherence to therapy is uncertain [22].

Recently, we have suggested that screening and constant monitoring of LTBI during TNF-α blocker therapy are determinants for continued treatment of PsA, in order to avoid risks of reactivation of tubercular disease. We have proposed that PsA patients with LTBI under anti-TNF-α therapy should undergo clinical and laboratory standard evaluation every 3 months and a chest radiograph every 6 months. Even patients negative for TST and IGRA should undergo clinical and laboratory standard evaluation every 3 months, but they could perform chest radiograph and IGRA every 12 months. At any time, in case of fever and symptoms and/or signs of suspected TB reactivation, they should stop biologic therapy and perform a chest X-ray and other appropriate diagnostic and therapeutic measures [15]. In conclusion, our experience suggests that anti-TNF-α treatment is effective and safe in PsA patients with concomitant LTBI. Therefore, neither LTBI nor chemoprophylaxis seems to influence the course of anti-TNF-α therapy.

Moreover, an accurate diagnosis and management of LTBI could reduce the risk of reactivation of tuberculosis. Further studies are needed to clarify the controversial question of the monitoring of LTBI patients on biologic therapy who show TST/IGRA positive conversion. At present, our approach is to treat and monitor them as patients with LTBI at enrollment.
